# Do Intravenous N-Acetylcysteine and Sodium Bicarbonate Prevent High Osmolal Contrast-Induced Acute Kidney Injury? A Randomized Controlled Trial

**DOI:** 10.1371/journal.pone.0107602

**Published:** 2014-09-25

**Authors:** Antonio Jose Inda-Filho, Adriano Caixeta, Marcia Manggini, Nestor Schor

**Affiliations:** 1 Divisão de Nefrologia, Hospital Universitário de Brasília, Universidade de Brasília, Brasília, DF, Brazil; 2 Cardiologia, Hospital Israelita Albert Einstein, São Paulo, SP, Brazil; 3 Cardiologia, Hospital Universitário de Brasília, Universidade de Brasília, Brasília, DF, Brazil; 4 Pós Graduação em Nefrologia, Universidade Federal de São Paulo, São Paulo, SP, Brazil; University of São Paulo State - Botucatu School of Medicine - UNESP, Brazil

## Abstract

**Background:**

N-acetylcysteine (NAC) or sodium bicarbonate (NaHCO_3_), singly or combined, inconsistently prevent patients exposed to radiographic contrast media from developing contrast-induced acute kidney injury (CI-AKI).

**Objective:**

We asked whether intravenous isotonic saline and either NaHCO_3_ in 5% dextrose or else a high dose of NAC in 5% dextrose prevent CI-AKI in outpatients exposed to high-osmolal iodinated contrast medium more than does saline alone.

**Methods:**

This completed prospective, parallel, superiority, open-label, controlled, computer-randomized, single-center, Brazilian trial (NCT01612013) hydrated 500 adult outpatients (214 at high risk of developing CI-AKI) exposed to ioxitalamate during elective coronary angiography and ventriculography. From 1 hour before through 6 hours after exposure, 126 patients (group 1) received a high dose of NAC and saline, 125 (group 2) received NaHCO_3_ and saline, 124 (group 3) received both treatments, and 125 (group 4) received only saline.

**Results:**

Groups were similar with respect to age, gender, weight, pre-existing renal dysfunction, hypertension, medication, and baseline serum creatinine and serum cystatin C, but diabetes mellitus was significantly less prevalent in group 1. CI-AKI incidence 72 hours after exposure to contrast medium was 51.4% (257/500), measured as serum creatinine > (baseline+0.3 mg/dL) and/or serum cystatin C > (1.1· baseline), and 7.6% (38/500), measured as both serum creatinine and serum cystatin C > (baseline+0.3 mg/dL) or > (1.25 · baseline). CI-AKI incidence measured less sensitively was similar among groups. Measured more sensitively, incidence in group 1 was significantly (p<0.05) lower than in groups 2 and 3 but not group 4; adjustment for confounding by infused volume equalized incidence in groups 1 and 3.

**Conclusion:**

We found no evidence that intravenous isotonic saline and either NaHCO_3_ or else a high dose of NAC prevent CI-AKI in outpatients exposed to high osmolal iodinated contrast medium more than does saline alone.

**Trial Registration:**

ClinicalTrials.gov NCT01612013.

## Introduction

Radiographic contrast media administered intravenously induce acute kidney injury (CI-AKI). Patients with CI-AKI experience an increase in the risk of mortality and in the cost and duration of hospitalization [1]–[5]. Despite advances in understanding the physiopathology of CI-AKI, the incidence of CI-AKI worldwide is significant and increasing because the use of contrast media is increasing [6]. For example, contrast media was used in an estimated 1 million percutaneous coronary interventions in the United States in 2010 [7]. Modern radiology units have abandoned the use of high-osmolal contrast media in patients with chronic kidney disease because the associated risk of inducing CI-AKI is high. But for economic reasons and because it is not clear whether the risk of developing CI-AKI is clinically important for the general population of patients, many public hospitals in Brazil and worldwide continue to use such media.

Contrast media is thought to induce the CI-AKI syndrome through a variety of mechanisms, including those that form and concentrate toxins–free radicals and acidity–in renal tubules [1], [8], [9]. Yet most clinical trials of the effect of renal detoxicants on CI-AKI incidence have tested only small homogeneous samples of patients, at high risk of developing CI-AKI due to affliction by diabetes mellitus or renal dysfunction, who were exposed to low- or iso-osmolal contrast media. In these patients, intravenous hydration with isotonic saline decreased the incidence of CI-AKI reliably, but hydration supplemented with the detoxicants N-acetylcysteine (NAC) or sodium bicarbonate (NaHCO_3_) further decreased incidence only inconsistently [10]–[17].

We hypothesized that CI-AKI-inducing mechanisms responsive to NAC or NaHCO_3_ treatment would be more evident in a large heterogeneous sample of outpatients exposed to high-osmolal contrast media. Our objective was to determine whether intravenously hydrating outpatients with isotonic saline supplemented with NaHCO_3_ or a high dose of NAC would protect them from developing CI-AKI within 72 hours after exposure to high-osmolal contrast medium more than would hydrating them with saline alone. We describe a completed single-center randomized controlled trial of 500 outpatients exposed to the high-osmolal contrast agent ioxitalamate during elective coronary angiography or ventriculography in which we compared the efficacy of four treatment strategies for preventing CI-AKI.

## Methods

The protocol for this trial and supporting CONSORT checklist are available as supporting information; see Checklist S1 and Protocol S1.

### Population and Study Protocol

We conducted this prospective, open-label, randomized, parallel-assignment, active-comparator, superiority, controlled trial (registered as NCT01612013 at ClinicalTrials.gov, http://clinicaltrials.gov/ct2/show/study/NCT01612013) at a single hospital center in Brazil between January 2007 and May 2009 in accordance with the principles of good clinical practice and the Declaration of Helsinki. The Research and Ethics Committee of Catholic University of Brasilia approved of the study protocol and all patients included in this trial gave informed written consent.

All outpatients 18 years of age and older scheduled for elective coronary angiography or ventriculography were eligible for inclusion in this study. A nurse, blinded to the trial hypothesis and from whom the random sequence of allocation to treatment was concealed, enrolled 500 eligible outpatients, excluding those who: were less than 18 years of age, received an iodinated contrast medium intravascularly within 30 days before evaluation for inclusion, received emergency coronary catheterization, experienced pulmonary edema or acutely decompensated congestive heart failure, were using nonsteroidal anti-inflammatory drugs or metformin at the time of the study, or declined to participate in the trial. No patient received theophylline, dopamine, or mannitol during the study.

A second nurse, blinded to the trial hypothesis and from whom enrollment information was concealed, in a room apart from that used for enrollment, prepared the medications and randomly allocated by computer without restriction each of 500 hundred outpatients satisfying the inclusion/exclusion criteria of this trial to one of four parallel treatment groups: NAC plus saline (group 1, n = 126), NaHCO_3_ plus saline (group 2, n = 125), NAC and NaHCO_3_ plus saline (group 3, n = 124), or saline (standard of care control group 4, n = 125).

Coronary angiography and ventriculography were performed according to standard protocols. Upon enrollment, all patients were instructed to avoid use of diuretic drugs for 48 hours before and after these procedures. All patients received the ionic, high-osmolality (2130 mOsm/kg, viscosity 7.5 mPa•s) contrast agent ioxitalamate (350 mg/mL iodine, Telebrix 35, Guerbet, Brazil). The dose of contrast medium administered, clinical management of the patient, and all adjunctive drug therapies were left to the discretion of the attending cardiologist.

### Medications

Patients received medications intravenously 60 minutes immediately before, during, and 6 hours immediately after contrast medium was administered. NAC (Flucistein, 100 mg/mL, Neo Química, Brazil) in 500 mL of 5% dextrose was given in bolus at 150 mg/(kg•h) before contrast medium was administered, then at 50 mg/(kg•h). NaHCO_3_, prepared by mixing 150 mEq (15 ampoules) of sodium bicarbonate (8.4%, Equiplex, Brazil) with 1 L of 5% dextrose, was given in bolus at 3.5 mL/(kg•h) before contrast medium was administered, then at 1.18 mL/(kg•h). Saline (0.9%, isotonic) was given intravenously at 1 mL/(kg•h).

### Creatinine and Cystatin C Measurements

Blood samples were obtained at baseline (before medication and contrast medium were infused and one hour before angiography or ventriculography) and at 24, 48, and 72 hours after contrast medium was administered.

Serum levels of creatinine (sCr) measured by the colorimetric method of Jaffe [18], and of cystatin C (sCys C) measured by an immunonephelometric method (Dade Behring, Marburg, Germany [19]), were used as biomarkers of the decreased renal glomerular filtration associated with CI-AKI. We estimated glomerular filtration rates (eGFR) in two ways: 1) 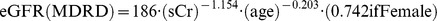, using sCr as a biomarker and the 4-variable Modification of Diet in Renal Disease formula (eGFR MDRD) as a model [20], and 2) 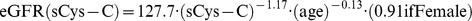, using sCys C as a biomarker [21].

### End Points and Definitions

The primary end point of this study was the development of CI-AKI between baseline and 72 hours after administration of contrast medium. Because the diagnostic definition of CI-AKI is evolving [22], [23], this study used two definitions of CI-AKI: 1) more sensitively, as 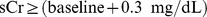 and/or 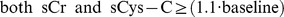; and 2) less sensitively, as 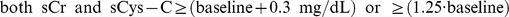. The secondary end point was the development of CI-AKI in a subgroup of high-risk patients with diabetes or pre-existing renal dysfunction, defined as a calculated baseline creatinine clearance <60 ml/min/1.73 m^2^ indicative of chronic kidney disease [1].

### Statistical Analysis

Values of continuous variables were summarized as mean (standard deviation). Values of categorical variables were summarized as frequency (percentage). P-values <0.05 were considered statistically significant.

A two-sided chi-square test (χ^2^ = 10.9050, df = 3) indicated that a sample size of 500 patients (125 patients per group) would have 80% power at a type I error probability of 0.05 to detect a statistically significant difference among the four groups and to detect a clinically meaningful 75% reduction of CI-AKI in treatment group 3. For this power calculation, the incidence of CI-AKI in control group 4 was estimated to be 15% because high-osmolal contrast medium was used in this study [1].

Homogeneity of the groups at baseline was evaluated with a chi square test for categorical variables (gender, diabetes mellitus, hypertension, medications, and pre-existing renal dysfunction) and with ANOVA for continuous variables (age, diastolic and systolic blood pressure, volume of contrast medium, infused volume, weight, serum creatinine, serum cystatin C, eGFR (MDRD), and eGFR (sCys C)). Multiple logistic regression analysis was used without covariables to compare the incidence of CI-AKI among the 4 groups and used with covariables (diabetes and infused volume; or the set of diabetes, infused volume, gender, age, and weight) to estimate a potential confounding effect of these covariables. A difference >10% in odds ratios calculated with and without a covariable was considered to indicate a confounding effect of the covariable.

To compensate for incomplete cases that were missing data, SAS software 9.3 was used to perform 4 multiple imputations, assuming that the data were missing at random, to achieve a relative imputation efficiency of at least 95%. For each variable and patient, each combined data point estimate from the 4 multiple imputations was calculated as the average of the 4 resulting complete-data estimates (Table S1).

Both an intent-to-treat analysis (n = 500) using imputed data and a per-protocol analysis (n = 425) using only the data of complete cases were performed using the software SPSS 15.0 (SPSS Corp., Chicago, Illinois, USA) for Windows.

The authors had full access to, and took full responsibility for the integrity of, the data. All authors have read and agreed with the manuscript as written.

## Results

### Patient Population and Baseline Characteristics

**Figure 1:**
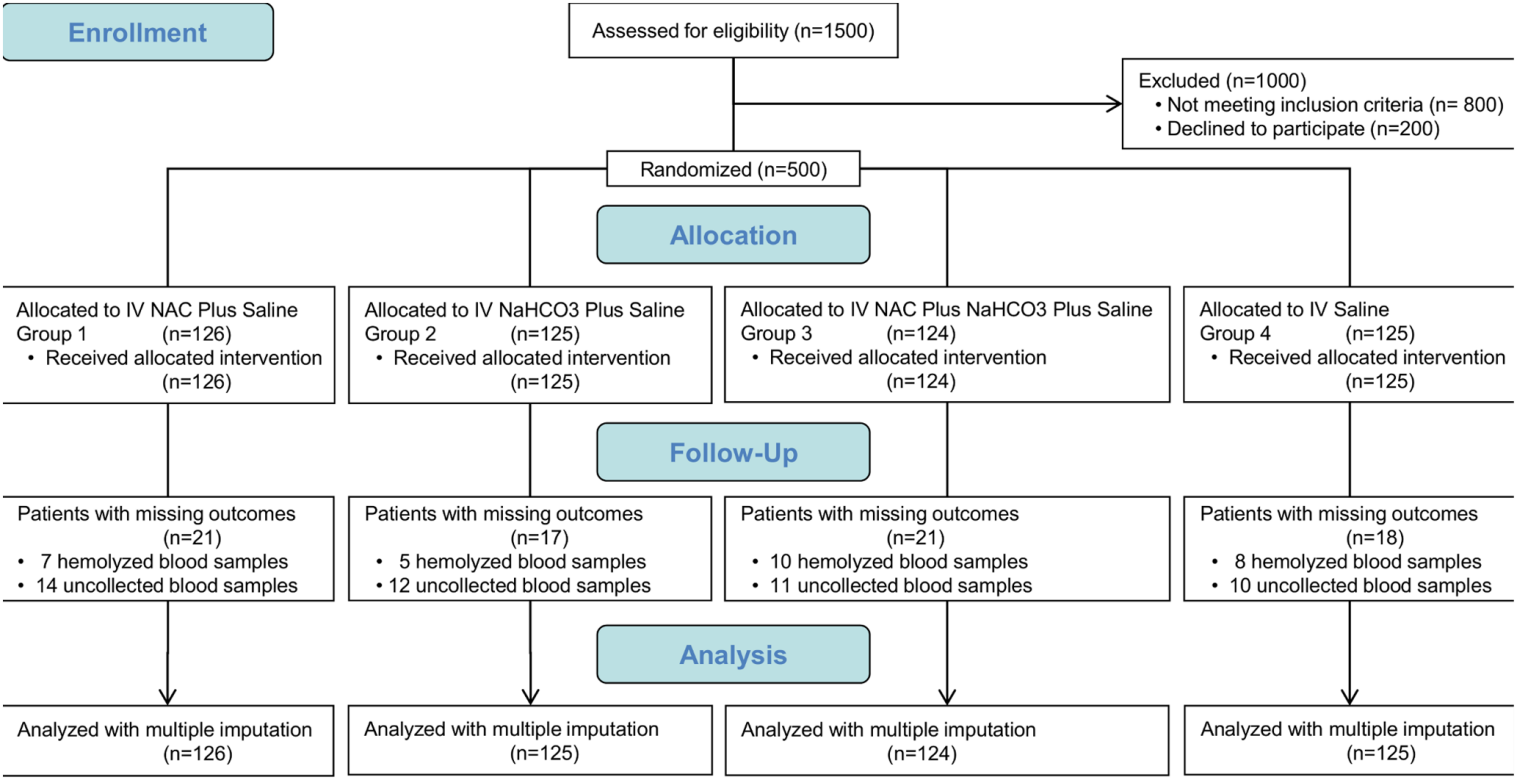
Flow of 500 outpatients through a parallel trial of four treatments for preventing CI-AKI. Practice of the trial design. Abbreviations: CI-AKI (contrast-induced acute kidney injury), IV (intravenous), NAC (N-acetylcysteine), NaHCO3 (sodium bicarbonate).

Of the 500 outpatients randomized to 4 treatment groups and exposed to contrast medium, 15% (75 of 500) were missing both sCr and sCys C data for some time points and 0.4% (2 of 500) were missing all sCr data (baseline, 24, 48 and 72 hours; Figure 1). The proportions of missing data were equivalent among all 4 groups, with no evidence of differential loss according to assigned treatment. Data was imputed to compensate for incomplete cases (Table S1).

Baseline values of patient traits in the 4 groups were well matched, except the prevalence of diabetes mellitus was significantly lower in group 1 than in groups 2 and 3 but not group 4 (Tables 1, S2, S3). Half of the patients were at high risk for developing CI-AKI, including 42.8% (214 of 500) who had pre-existing diabetes or renal dysfunction and an additional 7.2% (36 of 500) who received a large volume (more than 140 mL) of contrast medium.

**Table 1:** Baseline Traits and Infused Volumes of 500 Outpatients Hydrated After Exposure to Contrast Medium.

Baseline Characteristic, Mean(SD), n(%)	Trait	Group 1 (n = 126)	Group 2 (n = 125)	Group 3 (n = 124)	Group 4 (n = 125)	Total (n = 500)	Baseline Characteristic, Mean(SD), n(%)
	Age, years	59.2 (11.4)	59.1 (13.0)	58.6 (10.8)	60.5 (11.3)	59.4 (11.6)	
Gender	Male	78 (61.9)	70 (56)	82 (66.1)	73 (58.4)	303 (60.6)	0.386
	Female	48 (38.1)	55 (44)	42 (33.9)	52 (41.6)	197 (39.4)	0.386
Blood chemistry, mg/dL	Serum creatinine	1.00 (0.25)	1.00 (0.24)	1.07 (0.31)	1.04 (0.41)	1.03 (0.31)	0.203
	Serum cystatin C	0.88 (0.25)	0.85 (0.25)	0.93 (0.32)	0.95 (0.35)	0.90 (0.3)	0.051
Blood pressure, mm Hg	Systolic	135.9 (22.7)	135.2 (16.9)	136.4 (22.0)	137.7 (22.5)	136.3 (21.1)	0.822
	Diastolic	79.2 (12.3)	78.0 (11.0)	79.7 (11.7)	77.6 (12.6)	78.6 (11.9)	0.463
Estimated glomerular filtration rate, mL/min	eGFR (MDRD)	78.5 (18.7)	76.1 (17.9)	75.1 (21.5)	76.6 (23.1)	76.6 (20.4)	0.673
	eGFR (sCys C)	93.8 (32.7)	98.0 (39.4)	93.6 (50.2)	89.5 (34.6)	93.8 (39.7)	0.486
CI-AKI risk factor	Diabetes mellitus	18 (14.3)	31 (24.8)	25 (20.2)	36 (28.8)	110 (22.0)	0.036^a^
	Hypertension	53 (42.1)	51 (40.8)	61 (49.2)	56 (44.8)	221 (44.2)	0.554
	Renal dysfunction	22 (20.8)	24 (22.2)	27 (26.0)	31 (29.0)	104 (24.5)	0.496
Concomitant medication	ACE inhibitor	32 (25.4)	35 (28.0)	40 (32.3)	39 (31.2)	146 (29.2)	0.621
	ARB	7 (5.6)	10 (8.0)	10 (8.1)	12 (9.6)	39 (7.8)	0.69
	CCB	18 (14.3)	16 (12.8)	14 (11.3)	12 (9.6)	60 (12.0)	0.696
	B-blocker	34 (27.0)	38 (30.4)	39 (31.5)	37 (29.6)	148 (29.6)	0.883
	Statin	21 (16.7)	22 (17.6)	30 (24.2)	28 (22.4)	101 (20.2)	0.376
Infused volume, mL	Ioxitalamate	93.8 (34.2)	88.6 (30.9)	90.6 (33.6)	88.0 (25.1)	90.2 (31.1)	0.447
	Treatment	1482.7 (114.7)	1498.8 (129.7)	2494.7 (118.9)	482.1 (102.0)	1487.5 (720.4)	0

Description of outpatients in the intent-to-treat sample (n = 500) randomized to treatment. Groups were compared by using a chi square test for categorical variables and ANOVA for continuous variables.

a χ^2^ = 8.553, df = 3, 2-tailed P-value. Treatment: group 1 (N-acetylcysteine plus saline; NAC), group 2 (sodium bicarbonate plus saline; NaHCO_3_), group 3 (N-acetylcysteine plus sodium bicarbonate plus saline; NAC+NaHCO_3_), group 4 (saline). Abbreviations: ACE (angiotensin-converting enzyme), ARB (angiotensin II receptor blocker), B-blocker (beta-adrenergic blocking agent), CCB (calcium channel blocker), CI-AKI (contrast-induced acute kidney injury), eGFR (MDRD) (glomerular filtration rate estimated with a Modification of Diet in Renal Disease formula), eGFR (sCys C) (glomerular filtration rate estimated with a serum cystatin C formula).

### Incidence of CI-AKI

**Figure 2:**
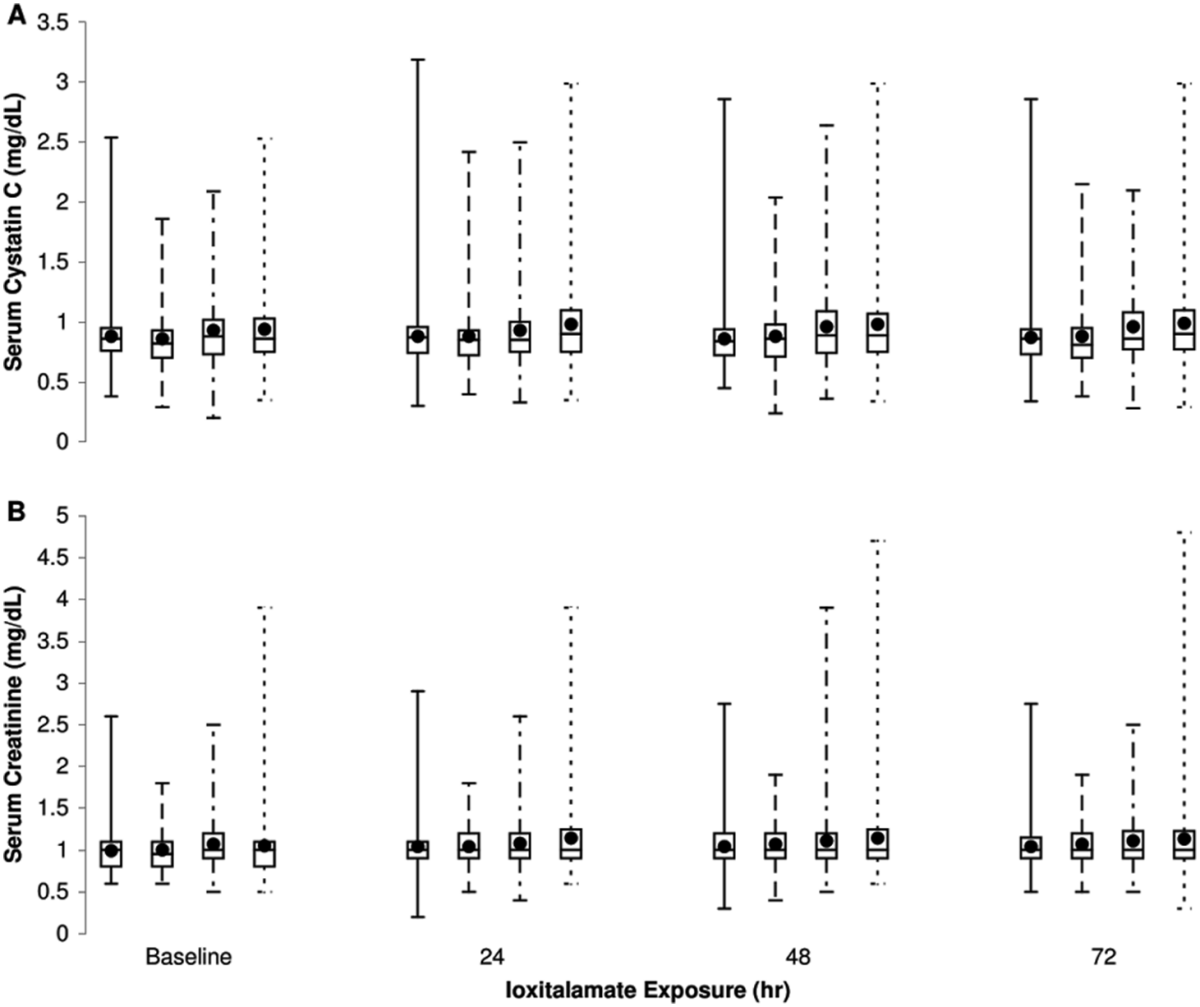
Change in serum creatinine and cystatin C levels of 500 outpatients exposed to ioxitalamate. Mean of serum creatinine (panel A) and serum cystatin C (panel B) concentrations before (baseline) and after administration of ioxitalamate, according to treatment group. Treatments: NAC (N-acetylcysteine plus saline; group 1), NaHCO3 (sodium bicarbonate plus saline; group 2), NAC+NaHCO3 (N-acetylcysteine plus sodium bicarbonate plus saline; group 3), saline (group 4). Means among the treatment groups did not differ significantly. Error bars indicate 1 standard deviation about the mean.

As expected [22], the observed incidence of CI-AKI depended on how CI-AKI was defined. Incidence among patients in the intent-to-treat sample was 51.4% (257 of 500) under the more sensitive definition of CI-AKI, but only 7.6% (38/500) under the less sensitive definition (Table 2). Average levels of sCr used to indicate CI-AKI were lowest at baseline and highest at 24 hours after exposure to contrast medium for groups 1 and 4 and at 48 hours for groups 2 and 3 (Figure 2, panel A). Average levels of sCys C used to indicate CI-AKI were lowest at baseline for groups 2, 3, and 4 and at 48 hours 4 for group 1; average levels were highest at baseline for group 1, at 24 hours for group 2, at 48 hours for group 3, and at 72 hours for group 4 (Figure 2, panel B).

**Table 2:** Incidence of CI-AKI in 500 Outpatients Hydrated After Exposure to Ioxitalamate.

Outcome	Indication	Group 1(n = 126)	Group 2(n = 125)	Group 3(n = 124)	Group 4(n = 125)	Total(n = 500)	P-value^a^
CI-AKI, n(%)	sCr ≥ (baseline+0.3 mg/dL) and/or sCysC≥(1.1•baseline)	49 (38.9%)	75 (60%)	72 (58.1%)	61 (48.8%)	257 (51.4%)	0.0032^b^
	both sCr and sCys C ≥(baseline+0.3 mg/dL) or≥(1.25•baseline)	9 (7.1%)	7 (5.6%)	8 (6.5%)	14 (11.2%)	38 (7.6%)	0.3493^c^

Efficacy of treatment in preventing outpatients in the intent-to-treat sample (n = 500) from developing CI-AKI after exposure to contrast medium.

a 2-tailed P-values resulting from chi square analysis;

b χ^2^ = 14.139, df = 3, post hoc analysis using the Bonferroni method indicated a significantly lower incidence of CI-AKI in Group 1 than in Group 2 (P-value = 0.006) and Group 3 (P-value = 0.024) but not Group 4 (P-value = 0.876);

c χ^2^ = 3.289; df = 3. Abbreviations: CI-AKI (contrast-induced acute kidney injury), sCr (serum creatinine), sCys C (serum cystatin C). Treatments are described in the legend of Table 1.

Under the less sensitive definition of CI-AKI, the incidence of CI-AKI among the four treatment groups in the intent-to-treat sample was uniform; but under the more sensitive definition, the incidence was significantly lower in group 1 than in groups 2 and 3 but not group 4 (Table 2). Of the conditions tested (age, diabetes, gender, infused volume, and weight), only the effect of infused volume appeared to be confounded with the effect of treatment (Table 3). Adjusting for that confounding effect equalized the effect of treatment in groups 1 and 3 but left the difference between groups 1 and 2 unchanged. We found no evidence that diabetic patients with pre-existing renal dysfunction were at increased risk of developing CI-AKI (Table 3).

**Table 3:** Multiple logistic regression analysis of potential confounding.

Sample	Model^a^	Predictor^b^	OddsRatio	Confidence Interval(95%)	P-value^c^	Difference in Odds Ratios (%)^d^
Total	1(unadjusted)	NAC(reference)	–	–	–	–
		NaHCO_3_	2.36	1.42; 3.91	**0.001**	–
		NAC + NaHCO_3_	2.18	1.31; 3.61	**0.003**	–
		Saline only	1.5	0.91; 2.47	0.114	–
	2 (adjusted only for infused volume)	NAC(reference)	–	–	–	–
		NaHCO_3_	2.33	1.4; 3.87	**0.001**	1.27
		NAC + NaHCO_3_	0.97	0.19; 4.99	0.97	**55.5**
		Saline only	3.34	0.65; 17.05	0.147	**-122.67**
		Infusedvolume	1	1; 1	0.31	–
	3 (adjusted for three predictors)	NAC (reference)	–	–	–	–
		NaHCO_3_	2.36	1.36; 4.1	**0.002**	0
		NAC + NaHCO_3_	1.39	0.18; 10.86	0.752	**36.24**
		Saline only	1.65	0.2; 13.26	0.64	**-10**
		Infusedvolume	1	1; 1	0.789	–
		Diabetes mellitus	1.17	0.73; 1.9	0.511	–
		Renal disease	1.02	0.65; 1.61	0.918	–
High Risk^e^	4 (unadjusted)	NAC (reference)	–	–	–	–
		NaHCO_3_	0.66	0.28; 1.53	0.329	–
		NAC + NaHCO_3_	1.49	0.69; 3.23	0.307	–
		Saline only	1.01	0.45; 2.27	0.976	–
	5 (adjusted only for infused volume)	NAC(reference)	–	–	–	–
		NaHCO_3_	1.5	0.08; 28.22	0.786	**-127.27**
		NAC + NaHCO_3_	3.51	0.17; 70.99	0.414	**-135.57**
		Saline only	5.27	0.02; 1537.05	0.566	**-421.78**
		Infusedvolume	1	1; 1	0.565	–
	6 (adjusted for three predictors)	NAC (reference)	–	–	–	–
		NaHCO_3_	2.23	0.88; 5.61	0.09	**-237.88**
		NAC + NaHCO_3_	1.16	0.05; 25.99	0.924	**22.15**
		Saline only	1.35	0.06; 31.48	0.851	**-33.66**
		Infused volume	1	1; 1	0.98	–
		Diabetes mellitus	1.72	0.7; 4.22	0.239	–
		Renal disease	1.54	0.61; 3.92	0.364	–

Adjustment for potential confounding of the effect of infused volume and of baseline diabetes and renal disease on induction of CI-AKI by ioxitalamate in the intent-to-treat (n = 500) and high risk (n = 250) samples. sCr (serum creatinine mg/dL); sCys C (serum cystatin C; mg/dL). Definition of CI-AKI (contrast-induced acute kidney injury): sCr≥(baseline+0.3 mg/dL) and/or both sCr and sCys C≥(baseline · (1+10%)).

a Regression models adjusted or unadjusted for potential confounding by infused volume and by baseline diabetes and renal disease.

b Predictor (all treatments included saline): NAC (N-acetylcysteine), NaHCO_3_ (sodium bicarbonate), saline alone; infused volume (total volume of oxitalamate plus treatment administered intravenously).

c bolded 2-tailed P-values were considered statistically significant and refer to the null hypothesis that the odds ratio = 1.

d Difference in odds ratios (%) = (100 · ((Unadjusted odds ratio) – (Adjusted odds ratio))/(Unadjusted odds ratio)); a difference of at least 10% (bolded value) was considered evidence of confounding.

e Patients at high risk of developing CI-AKI due to affliction with diabetes mellitus or renal dysfunction at baseline or due to receiving >140 mL of ioxitalamate.

### Adverse events

No patient developed renal failure requiring temporary dialysis. No patient was withdrawn from the study, although adverse events occurred in 5 patients treated with NAC (rash, nausea, headache, and bronchospasm) and in 4 patients treated with NaHCO_3_ (pulmonary edema).

Similar results were obtained with a per-protocol analysis.

## Discussion

The main finding of this trial, like that of some [24]–[33] trials, is that isotonic saline supplemented with NAC or NaHCO_3_ provides outpatients exposed to contrast medium no more protection from developing CI-AKI than does isotonic saline alone. But the lower incidence of CI-AKI (defined more sensitively) in group 1 than in group 2 suggests that a larger trial of outpatients might detect a small but clinically meaningful benefit of NAC treatment, consistent with the finding of other trials [34]–[41]. Preclinical studies suggest that saline hydration, NAC, and NaHCO_3_ may protect against the damaging effects of contrast media by inhibiting the formation, accumulation, or concentration of free radicals responsible for oxidative damage to renal tubules [22], [42], [43].

We observed a higher incidence of CI-AKI than have others [1], [22], [23], which might be explained in three ways. 1) Specialized diagnostic definitions of CI-AKI–for example, one for patients with diseased kidneys and another for patients with healthy kidneys–may be needed for consistency among trials. The definition of CI-AKI as an increase in sCr [44] or sCys C [45] above baseline was developed in trials of patients with diseased kidneys [22]. If that definition is less specific for patients with healthy kidneys, then using it may overestimate the incidence of CI-AKI in trials such as ours that include a large proportion of such patients. 2) A more reliable biomarker may be needed. A transient increase of popular biomarkers to levels indicating CI-AKI can result from conditions unrelated to CI-AKI: transient hypotension or variations in dietary intake and hydration affect sCr levels [22], [46], [47] and thyroid dysfunction affects sCys C levels [48], [49]. If those conditions were more pervasive in our single-center trial than in other trials, then our choice of biomarkers would have led us to overestimate the incidence of CI-AKI. 3) Our observations suggest that high-osmolal contrast medium may be toxic to all patients. Unlike most trials, which expose patients with pre-existing renal dysfunction to only low- or iso-osmolal contrast medium, our trial exposed all patients to high-osmolal contrast medium [22]. We observed both: an equal incidence of CI-AKI in low-risk diabetic patients without pre-existing renal dysfunction and in high-risk diabetic patients with pre-existing renal dysfunction; and an overall incidence (51.4%, 257/500) that exceeded the percentage of patients at high risk for developing CI-AKI (50%, 250/500). We therefore recommend abandoning the use of high-osmolal contrast media worldwide.

### Limitations

The results of this study should not be generalized to patients, such as those with chronic kidney disease, who are exposed to hypo- or iso-osmolal contrast media. High-osmolal contrast media are more nephrotoxic to patients with pre-existing renal failure than are low- or iso-osmolal media [50], [51]. All outpatients in our trial were recruited from one hospital and received only high-osmolal contrast medium. Half of them had normal kidney function and were not at high risk for developing CI-AKI.

Optimal methods of administering medication to treat CI-AKI have not yet been identified. Total infused volume appeared to be a confounding factor in our study. And although the Kidney Disease Improving Global Outcomes and European Renal Best Practice guideline recommends oral administration of NAC to patients at risk of developing CI-AKI [52], recent findings suggest that orally administered NAC provides no therapeutic benefit [53]–[56].

We hypothesized that a large trial of diverse outpatients might detect mechanisms of CI-AKI development that would respond to NAC or NaHCO_3_ treatments. Even though this trial failed to detect such mechanisms, our hypothesis might still be useful. Our trial was underpowered due to a flaw in our calculation of statistical power. Rather than assume a large effect of treatment (a 75% decrease in the incidence of CI-AKI), we should have assumed a smaller effect no greater than 40% [39], [57], [58]. A post hoc analysis showed that, under the more sensitive definition of CI-AKI for a sample size of 500 patients and a 5% level of significance, the power to detect a statistically significant difference from the control group 4 was 27% (group 2), 21% (group 1), and 18% (group 3); under the less sensitive definition of CI-AKI, the power was 21% (group 2), 10% (group 1), and 14% (group 3).

This trial, like most others, suffered from loss to follow up. Everyone agrees that missing data should be minimized because it precludes the use of standard statistical techniques for intent-to-treat analysis and because per-protocol analysis can substantially bias estimates of treatment effects in superiority trials [59]–[61] like ours. But not everyone agrees on how to address missing data. The statistical technique of multiple imputation (MI) that we used to compensate for missing data in this study is widely accepted and usually allows analysis of data from all patients randomized to treatment without introducing bias [62]–[64].

We were unable to distinguish variations in sCr and sCys C associated with exposure to ioxitalamate from variations not associated with exposure [22], [46], [65] because our trial lacked a placebo control group that was not exposed to ioxitalamate.

## Conclusion

Analysis of the results of this prospective, randomized, single-center, controlled trial revealed no evidence that intravenously hydrating outpatients with isotonic saline supplemented with NaHCO_3_ or a high dose of NAC protected them from developing CI-AKI within 72 hours after exposure to high-osmolal contrast medium more than did hydrating them with saline alone.

## Supporting Information

Table S1 **Imputed data.** SAS software 9.3 was used to perform 4 multiple imputations for each missing datum. Each combined data point estimate was calculated as the average of the 4 resulting complete-data estimates.
(DOC)

Table S2 **Baseline summary statistics.** P-values of an ANOVA model for the total study group and the group at high risk for developing CI-AKI.
(DOC)

Table S3 **ANOVA analysis of baseline variables.** A bolded value denotes a P-value >0.05.
(DOC)

Checklist S1 CONSORT 2010 checklist of information to include when reporting a randomised trial.
(PDF)

Protocol S1 Study protocol.
(PDF)
