# Supplementary material for: Do Intravenous N-Acetylcysteine and Sodium Bicarbonate Prevent High Osmolal Contrast-Induced Acute Kidney Injury? A Randomized Controlled Trial
Source: PLoS One. 2014 Sep 25;9(9):e107602. doi: 10.1371/journal.pone.0107602 (PMC4177831; doi:10.1371/journal.pone.0107602)
Supplement: Table S1 — Imputed data. SAS software 9.3 was used to perform 4 multiple imputations for each missing datum. Each combined data point estimate was calculated as the average of the 4 resulting complete-data estimates. (DOC) [file pone.0107602.s001.doc]

**Table S1. Imputed data.**

| **Patient** | **Group** | **sCys C (mg/dL)** | | | | **sCr (mg/dL)** | | | |
| --- | --- | --- | --- | --- | --- | --- | --- | --- | --- |
|  |  | **Baseline** | **24 hours** | **48 hours** | **72 hours** | **Baseline** | **24 hours** | **48 hours** | **72 hours** |
| 11 | 3 | 1.26 | 1.37 | 1.26 | 1.42 | 1.18 | 1.05 | 1.15 | 1.23 |
| 12 | 4 | 1.03 | 1.05 | 1.11 | 1.12 | 1.18 | 1.45 | 1.30 | 1.38 |
| 13 | 1 | 0.82 | 0.78 | 0.92 | 0.96 | 1.03 | 1.10 | 1.08 | 1.14 |
| 16 | 3 | 0.77 | 0.87 | 1.11 | 1.05 | 0.95 | 1.00 | 1.08 | 1.05 |
| 17 | 4 | 0.88 | 0.98 | 0.97 | 0.99 | 1.03 | 1.13 | 1.15 | 1.00 |
| 18 | 1 | 0.80 | 0.68 | 0.81 | 0.76 | 0.78 | 0.88 | 0.88 | 0.90 |
| 20 | 3 | 0.89 | 0.94 | 0.96 | 0.95 | 1.03 | 1.23 | 1.35 | 1.23 |
| 21 | 4 | 1.10 | 1.07 | 1.07 | 1.09 | 1.13 | 1.23 | 1.18 | 1.25 |
| 23 | 2 | 0.83 | 0.84 | 0.91 | 0.98 | 1.13 | 0.95 | 0.98 | 1.13 |
| 26 | 1 | 1.02 | 1.08 | 1.09 | 1.11 | 0.93 | 0.98 | 1.03 | 0.98 |
| 33 | 4 | 0.94 | 0.95 | 0.81 | 0.88 | 1.15 | 0.95 | 0.93 | 1.00 |
| 35 | 2 | 0.77 | 0.74 | 0.71 | 0.68 | 0.83 | 0.91 | 0.95 | 0.88 |
| 36 | 3 | 0.68 | 0.83 | 0.88 | 0.87 | 0.90 | 0.88 | 0.93 | 0.93 |
| 40 | 3 | 0.89 | 0.87 | 1.02 | 1.04 | 1.00 | 1.10 | 1.15 | 1.23 |
| 50 | 1 | 0.86 | 0.84 | 0.65 | 0.72 | 0.93 | 0.78 | 0.85 | 1.03 |
| 59 | 2 | 0.65 | 0.73 | 0.75 | 0.71 | 0.90 | 1.15 | 1.03 | 1.05 |
| 66 | 1 | 0.92 | 0.94 | 0.82 | 0.94 | 1.25 | 1.28 | 1.88 | 1.28 |
| 87 | 2 | 0.86 | 0.78 | 0.83 | 0.84 | 0.92 | 0.90 | 0.97 | 1.00 |
| 92 | 3 | 1.07 | 1.00 | 1.03 | 1.13 | 1.23 | 1.15 | 1.10 | 1.23 |
| 96 | 3 | 0.81 | 0.75 | 0.78 | 0.89 | 1.23 | 1.13 | 1.13 | 1.15 |
| 126 | 1 | 0.89 | 0.91 | 0.90 | 0.86 | 1.03 | 0.98 | 1.08 | 1.25 |
| 128 | 3 | 0.82 | 0.93 | 0.99 | 1.03 | 1.15 | 1.13 | 1.15 | 1.35 |
| 129 | 4 | 0.89 | 1.00 | 0.93 | 0.90 | 0.83 | 0.95 | 1.05 | 0.88 |
| 130 | 1 | — | — | — | — | 1.55 | 1.55 | 1.45 | 1.45 |
| 147 | 2 | 0.71 | 0.68 | 0.68 | 0.63 | 0.78 | 0.93 | 0.78 | 0.80 |
| 148 | 3 | 0.82 | 0.89 | 0.84 | 0.83 | 0.88 | 1.05 | 1.10 | 1.10 |
| 174 | 1 | 1.33 | 1.87 | 1.64 | 1.75 | 1.70 | 1.88 | 1.73 | 1.55 |
| 175 | 2 | 0.81 | 0.77 | 0.82 | 0.82 | 1.01 | 1.03 | 1.00 | 1.20 |
| 176 | 3 | 0.87 | 0.89 | 0.93 | 0.88 | 0.90 | 0.93 | 0.95 | 1.08 |
| 178 | 1 | 0.95 | 0.94 | 0.86 | 0.85 | 0.85 | 0.95 | 0.93 | 0.83 |
| 194 | 1 | 0.95 | 0.90 | 0.81 | 0.74 | 0.98 | 0.80 | 0.90 | 0.93 |
| 199 | 2 | 0.75 | 0.76 | 0.76 | 0.75 | 1.18 | 1.18 | 1.10 | 1.20 |
| 200 | 3 | 0.95 | 0.87 | 0.91 | 0.89 | 1.08 | 1.20 | 1.03 | 1.03 |
| 201 | 4 | 0.89 | 0.94 | 0.91 | 1.00 | 0.93 | 1.13 | 1.08 | 1.23 |
| 202 | 1 | 1.00 | 0.90 | 0.80 | 0.83 | 0.83 | 1.00 | 0.98 | 0.88 |
| 203 | 2 | 1.04 | 1.11 | 1.22 | 1.16 | 1.18 | 1.38 | 1.38 | 1.60 |
| 209 | 1 | 0.92 | 0.85 | 0.86 | 0.79 | 0.80 | 0.85 | 0.85 | 0.88 |
| 225 | 1 | 0.81 | 0.76 | 0.83 | 0.77 | 0.85 | 1.01 | 1.00 | 0.90 |
| 226 | 2 | 1.00 | 0.89 | 0.92 | 0.91 | 1.13 | 1.20 | 1.20 | 1.18 |
| 229 | 1 | 0.91 | 0.97 | 0.99 | 0.93 | 1.25 | 1.23 | 1.28 | 0.98 |
| 230 | 2 | 0.79 | 0.76 | 0.84 | 0.78 | 1.13 | 1.15 | 1.08 | 1.08 |
| 231 | 3 | 1.07 | 1.15 | 1.24 | 1.19 | 1.18 | 1.23 | 1.80 | 1.33 |
| 232 | 4 | 1.03 | 1.10 | 1.05 | 1.06 | 0.95 | 1.03 | 1.18 | 1.03 |
| 235 | 3 | 0.81 | 0.81 | 0.74 | 0.79 | 1.00 | 0.90 | 0.98 | 1.03 |
| 236 | 4 | 0.93 | 0.99 | 1.05 | 0.99 | 1.05 | 1.25 | 1.25 | 1.23 |
| 238 | 2 | 0.80 | 0.80 | 0.81 | 0.90 | 1.23 | 1.18 | 1.25 | 1.38 |
| 239 | 3 | — | — | — | — | 1.00 | 0.95 | 0.93 | 1.15 |
| 241 | 1 | 0.89 | 0.80 | 0.71 | 0.71 | 0.90 | 0.90 | 1.00 | 0.98 |
| 267 | 3 | 0.81 | 0.80 | 0.77 | 0.73 | 1.13 | 1.03 | 0.95 | 1.08 |
| 268 | 4 | 1.61 | 1.76 | 1.65 | 1.82 | 1.58 | 1.43 | 1.50 | 1.68 |
| 269 | 1 | 0.92 | 0.91 | 0.86 | 0.86 | 1.05 | 1.03 | 1.00 | 1.08 |
| 273 | 1 | 0.87 | 0.79 | 0.90 | 0.87 | 0.75 | 0.85 | 0.85 | 0.85 |
| 274 | 2 | 0.82 | 0.80 | 0.83 | 0.83 | 0.88 | 0.95 | 0.98 | 0.93 |
| 275 | 3 | 0.91 | 0.94 | 0.96 | 0.87 | 0.70 | 0.83 | 0.88 | 0.85 |
| 276 | 4 | 0.92 | 0.84 | 0.96 | 1.09 | 0.88 | 0.88 | 1.00 | 1.20 |
| 278 | 2 | 0.73 | 0.73 | 0.68 | 0.68 | 0.83 | 0.93 | 0.98 | 0.95 |
| 279 | 3 | 0.91 | 0.84 | 0.90 | 0.88 | 1.05 | 1.15 | 1.25 | 1.15 |
| 280 | 4 | 0.88 | 0.86 | 0.85 | 0.95 | 1.00 | 1.25 | 1.25 | 1.13 |
| 281 | 1 | 0.83 | 0.85 | 0.85 | 0.85 | 0.88 | 0.95 | 1.00 | 0.98 |
| 284 | 4 | 0.89 | 0.82 | 0.78 | 0.79 | 0.95 | 1.03 | 1.03 | 0.98 |
| 287 | 3 | 0.84 | 0.88 | 0.99 | 0.99 | 1.15 | 1.03 | 1.15 | 1.18 |
| 288 | 4 | 0.90 | 0.83 | 0.85 | 0.87 | 1.15 | 1.13 | 1.23 | 1.15 |
| 296 | 4 | 1.11 | 1.21 | 1.10 | 1.22 | 1.05 | 1.00 | 1.13 | 1.15 |
| 311 | 3 | 0.88 | 0.96 | 0.99 | 0.88 | 1.08 | 1.05 | 1.10 | 1.08 |
| 326 | 2 | 0.70 | 0.64 | 0.70 | 0.80 | 0.98 | 1.00 | 0.98 | 1.18 |
| 332 | 4 | 0.92 | 0.89 | 0.83 | 0.86 | 0.78 | 0.83 | 0.88 | 0.80 |
| 337 | 1 | 0.93 | 0.88 | 0.82 | 0.77 | 1.18 | 1.23 | 1.25 | 1.23 |
| 339 | 3 | 0.71 | 0.77 | 0.85 | 0.85 | 1.08 | 1.08 | 1.08 | 1.08 |
| 346 | 2 | 0.84 | 0.72 | 0.81 | 0.80 | 0.90 | 1.00 | 1.00 | 0.93 |
| 350 | 2 | 0.91 | 0.80 | 0.76 | 0.78 | 1.13 | 1.25 | 1.18 | 1.23 |
| 352 | 4 | 0.92 | 0.81 | 0.92 | 1.08 | 0.88 | 1.00 | 1.13 | 1.13 |
| 353 | 1 | 0.80 | 0.87 | 0.82 | 0.85 | 0.85 | 0.88 | 0.90 | 0.93 |
| 372 | 4 | 1.02 | 0.95 | 1.01 | 0.97 | 1.03 | 1.23 | 1.10 | 1.13 |
| 399 | 3 | 0.75 | 0.76 | 0.84 | 0.76 | 1.18 | 1.00 | 1.00 | 0.85 |
| 422 | 2 | 0.80 | 0.74 | 0.84 | 0.75 | 1.00 | 1.00 | 1.23 | 1.15 |
| 484 | 4 | 0.87 | 0.97 | 0.99 | 1.03 | 0.95 | 1.08 | 1.15 | 1.15 |
| 489 | 1 | 0.79 | 0.75 | 0.78 | 0.64 | 1.03 | 0.93 | 1.00 | 1.15 |
