# Supplementary material for: Do Intravenous N-Acetylcysteine and Sodium Bicarbonate Prevent High Osmolal Contrast-Induced Acute Kidney Injury? A Randomized Controlled Trial
Source: PLoS One. 2014 Sep 25;9(9):e107602. doi: 10.1371/journal.pone.0107602 (PMC4177831; doi:10.1371/journal.pone.0107602)
Supplement: Table S2 — Baseline summary statistics. P-values of an ANOVA model for the total study group and the group at high risk for developing CI-AKI. (DOC) [file pone.0107602.s002.doc]

Table S2. Baseline statistics.

|  |  | Group 1 CI-AKI | | Group 2 CI-AKI | | Group 3 CI-AKI | | Group 4 CI-AKI | | Group at High Risk of CI-AKI | | |
| --- | --- | --- | --- | --- | --- | --- | --- | --- | --- | --- | --- | --- |
| Trait | Statistic | Yes (n=46) | No (n=60) | Yes (n=70) | No (n=38) | Yes (n=61) | No (n=43) | Yes (n=53) | No (n=54) | Yes (n=74) | No (n=91) | P-value |
| Age | mean(SD) | 60.9(11.3) | 58.7(12.2) | 61.2(12.8) | 59(12.2) | 58.9(11.6) | 59(10.1) | 62.2(10.9) | 57.9(11.1) | 63.9(10.8) | 63.7(9.7) | 0.904 |
|  | median(range) | 60.5(39;82) | 60(21;83) | 58.5(28;79) | 58.5(28;79) | 61(33;85) | 60(30;74) | 60(40;86) | 59(33;80) | 65(40;86) | 63.5(33;83) |  |
| Blood pressure (diastolic) | mean(SD) | 81.5(11.9) | 77.8(12) | 78.2(11.2) | 75.4(10.6) | 79.7(12.2) | 77.8(10.3) | 78.6(13.4) | 75.4(12.5) | 79.2(12.2) | 76.8(13.4) | 0.215 |
|  | median(range) | 80(54;122) | 78(54;115) | 75.5(51;98) | 75.5(51;98) | 81(44;104) | 78(59;100) | 79(42;106) | 76(49;101) | 80(42;104) | 77(49;115) |  |
| Blood pressure (systolic) | mean(SD) | 141.2(26.9) | 134.9(18.2) | 136.5(16.9) | 131.3(15.7) | 136.3(20.5) | 133.8(25) | 137.2(20.8) | 135.4(24.8) | 140.2(22.3) | 138.7(24.8) | 0.684 |
|  | median(range) | 136.5(90;212) | 132(93;180) | 128.5(100;181) | 128.5(100;181) | 136(80;175) | 132(87;214) | 138(81;193) | 130(81;185) | 138(81;212) | 138(81;214) |  |
| Infused volume | mean(SD) | 1506.4(88.8) | 1498.2(82.5) | 1512.8(122.5) | 1498(75.7) | 2486.5(99.4) | 2526.6(112.5) | 497.6(109.9) | 470.6(74.7) | 1438.1(716.9) | 1447.6(766.2) | 0.935 |
|  | median(range) | 1504(1343;1756) | 1493.5(1322;1715.4) | 1490(1350;1686) | 1490(1350;1686) | 2469(2296.8;2819) | 2497(2294;2847) | 477.4(336;924) | 479.5(315;609) | 1502.6(336;2658) | 1493.5(315;2847) |  |
| Weight | mean(SD) | 72.3(12.7) | 71.2(11.8) | 73.3(17.5) | 71.1(10.8) | 69.5(14.2) | 75.2(16.1) | 71.1(15.7) | 67.2(10.7) | 72(16.4) | 71.7(14.2) | 0.889 |
|  | median(range) | 72(49;108) | 70.5(46;102.2) | 70(50;98) | 70(50;98) | 67(42.4;117) | 71(42;121) | 68.2(48;132) | 68.5(45;87) | 70(42.4;132) | 71.1(42;121) |  |
