# Supplementary material for: Do Intravenous N-Acetylcysteine and Sodium Bicarbonate Prevent High Osmolal Contrast-Induced Acute Kidney Injury? A Randomized Controlled Trial
Source: PLoS One. 2014 Sep 25;9(9):e107602. doi: 10.1371/journal.pone.0107602 (PMC4177831; doi:10.1371/journal.pone.0107602)
Supplement: Table S3 — ANOVA analysis of baseline variables. A bolded value denotes a P-value >0.05. (DOC) [file pone.0107602.s003.doc]

**Table S3. ANOVA analysis of baseline variables.**

| **Variable** | | **P-value** |
| --- | --- | --- |
| Age | Group | 0.894 |
|  | CI-AKI | 0.064 |
|  | CI-AKI X Group | 0.599 |
| Blood pressure (systolic) | Group | 0.543 |
|  | CI-AKI | 0.059 |
|  | CI-AKI X Group | 0.841 |
| Blood pressure (diastolic) | Group | 0.245 |
|  | CI-AKI | **0.013** |
|  | CI-AKI X Group | 0.946 |
| Weight | Group | 0.316 |
|  | CI-AKI | 0.800 |
|  | CI-AKI X Group | 0.079 |
| Infused volume (ml) | Group | **< 0.001** |
|  | Group 1 X Group 2 | 1.000 |
|  | Group 1 X Group 3 | **< 0.001** |
|  | Group 1 X Group 4 | **< 0.001** |
|  | Group 2 X Group 3 | **< 0.001** |
|  | Group 2 X Group 4 | **< 0.001** |
|  | Group 3 X Group 4 | **< 0.001** |
|  | CI-AKI | 0.800 |
|  | CI-AKI X Group | 0.079 |
