# Supplementary material for: Do Intravenous N-Acetylcysteine and Sodium Bicarbonate Prevent High Osmolal Contrast-Induced Acute Kidney Injury? A Randomized Controlled Trial
Source: PLoS One. 2014 Sep 25;9(9):e107602. doi: 10.1371/journal.pone.0107602 (PMC4177831; doi:10.1371/journal.pone.0107602)
Supplement: Protocol S1 — Study protocol. (PDF) [file pone.0107602.s005.pdf]

INSTITUTO DO CORÇÃO DO DISTRITO FEDERAL

Proposed appropriation of funds for medical research and/or experimental

Process InCor-DF n.________________

Title of the Project:
Contrast agent x acute reduction of renal function. Prevention: acetylcysteine and/or sodium bicarbonate

“The presentation of this cost spreadsheet is mandatory for all research projects to be developed in this institution, regardless of whether they have specific funding or not.”

This spreadsheet must be submitted along with the submission of the project for analysis by the Ethics Committee.

Name of the Chief Researcher: Antonio Jose de Almeida Inda Filho

Name of the Advisor (when there is one) _____________________________

E.mail: indafilho@hotmail.com

Telephones: 9982 4273; 345 0303; 344 7464

INSTITUTO DO CORÇÃO DO DISTRITO FEDERAL

Proposed appropriation of funds for medical research and/or experimental

Process InCor-DF n.________________

Name of the Chief Researcher: Antonio Jose de Almeida Inda Filho

Estimated period for the project: FROM 01/04/2005 UNTIL 31/12/2007

x Research with no specific funding **x Prospective Study**

Funded research **Prospective Study**

Overall listing of the expenses on the development of the project (sponsored or not)

Acquisition of Permanent National Material	(Form 1)	R$ 00,00
Purchase or consumption of permanent imported material	(Form 2)	R$ 00,00
Acquisition of consumables	(Form 3)	R$ 56478,00
Acquisition of imported consumables	(Form 4)	R$ 00,00
Expenses on hiring specific staff	(Form 5 e 6)	R$ 00,00
Transportation expenses paid by the project	(Form 7)	R$ 00,00
Expenses on laboratory tests	(Form 8)	R$ 63400,00
on radiology		R$ 00,00
on pathology		R$ 00,00
other tests		R$ 00,00
Individual expenses of volunteer	(Form 9)	R$ 00,00
Expenses on the acquisition of animals	(Form 10)	R$ 00,00
Expenses for maintenance of animals in vivarium	(Form 10)	R$ 00,00
Remuneration of researchers and third parties		R$ 00,00
Administrative value intended to Incor-DF		R$ 00,00
Other (mention) stationery and transport of test material		R$ 11992,00
TOTAL		R$ 131870,00
FUNDING SOURCE	Hospital of the Armed Forces and own funding	

Names of the professionals involved in the research	post
Antonio Jose de Almeida Inda Filho	Nephrologist
Marcia Manggini	Nurse
Adriano Caixeta	Hemodynamicist
Nestor Schor	Nephrologist

INSTITUTO DO CORÇÃO DO DISTRITO FEDERAL

Proposed appropriation of funds for medical research and/or experimental

Process InCor-DF n.________________

Place where the research will be conducted

Hospital ☐x InCor-DF ☐ other (mention) Hospital Santa Luzia and Hospital de Base

Laboratory (mention) Laboratory of the Hospital of the Armed Forces
Thesis ☐ no ☐ yes (☐ Master Degree ☐ Doctoral Degree)
Specialisation ☐ no ☐ yes

2

METHODOLOGY:

THE METHODOLOGY TO BE USED IN THE RESEARCH IS AN ANALYTICAL STUDY - CLINICAL, PROSPECTIVE, RANDOMIZED AND CONTROLLED TRIAL.

STUDY POPULATION:

INCLUSION CRITERIA:

IT WILL BE ELIGIBLE FOR INCLUSION IN THE STUDY 500 OUTPATIENTS WITH NORMAL RENAL FUNCTION OR CHRONIC RENAL FAILURE , WITH OR WITHOUT DIABETES MELLITUS , MALE OR FEMALE WHO ARE REFERRED TO THE DEPARTMENT OF HEMODYNAMIC OF THE INSTITUTION BY THEIR ASSISTANT DOCTORS, WITH INDICATION FOR EXAM – CARDIAC ANGIOGRAPHY COVERED BY THEIR HEALTH CARE INSURANCE, WHO GRANT INFORMED CONSENT AND WHO MEET THE FOLLOWING CRITERIA: AGE BETWEEN 18 AND 85 YEARS OLD, NORMAL OR INCREASED SERUM CREATININE (CREATININE >1.4 MG/DL AND 6,0 MG/DL). IT WILL BE PERFORMED ANAMNESIS AND PHYSICAL EXAMINATION IN ALL THOSE ELIGIBLE. IT WILL BE EMPHASIZED THE PRESENCE OR ABSENCE OF CHRONIC MEDICAL CONDITIONS, ESPECIALLY DIABETES MELLITUS, PERIPHERAL VASCULAR AND CEREBROVASCULAR DISEASE. ALL MEDICATIONS IN USE WILL BE RECORDED. PROLONGED USE OF STABLE DOSES OF CALCIUM-CHANNEL BLOCKERS, ANGIOTENSIN-CONVERTING ENZYME INHIBITORS, AND NONSTEROIDAL ANTI-INFLAMMATORY DIPYRIDAMOLE IS NOT AN EXCLUSION FACTOR. START, STOP OR CHANGE THE DOSE DURING THE STUDY WILL NOT BE PERMITTED.

EXCLUSION CRITERIA:

PATIENTS WHO ARE ALREADY ON DIALYSIS OR THOSE SUFFERING FROM AUTOIMMUNE DISEASES WILL BE EXCLUDED FROM THE STUDY, THOSE WHO HAVE SUFFERED AN ACUTE MYOCARDIAL INFARCTATION WITHIN THE PAST SIX MONTHS, SEVERE ALLERGIES, LIVER CIRRHOSIS, NEPHROTIC SYNDROME, SEVERE CHRONIC OBSTRUCTIVE VENTILATION DISORDER, BLOOD DYSCRASIAS, ONGOING PREGNANCY, CONGESTIVE CLASS IV (NYHA) HEART FAILURE, UNSTABLE ANGINA PECTORIS, (SYSTOLIC >220 mmHg) SEVERE UNCONTROLLED HYPERTENSION, HYPOTENSION (SYSTOLIC <80 mmHg) OR ANY OTHER CONDITION THAT, IN THE OPINION OF THE INVESTIGATOR, DETERMINE RISK TO THE PATIENT.

STUDY DESIGN:

THE SELECTED PATIENTS WILL RECEIVE NON-IONIC CONTRAST, MONOMERIC AND LOW OSMOLALITY AND WILL BE RANDOMLY ASSIGNED TO RECEIVE ACETYLCYSTEINE, SODIUM BICARBONATE, THE COMBINATION OF THESE TWO DRUGS OR CONTROL (0.9% SALINE SOLUTION).

WE WILL VISUALIZE THE STUDY AS FOLLOW:

GROUP A – ACETYLCYSTEINE GROUP

GROUP B – SODIUM BICARBONATE GROUP

GROUP C – ACETYLCYSTEINE GROUP/SODIUM BICARBONATE

GROUP D – CONTROL GROUP

THE DOSAGE OF EACH MEDICATION WILL BE THE FOLLOWING:

- Acetylcysteine: 150 mg/kg diluted in 500 ml of 5% glucose solution intravenously, 60 (sixty) minutes before exposure to contrast, and 50 mg/kg diluted in 500 ml saline solution (0.9%) intravenously for 6 hours after the exam.
- Sodium Bicarbonate: 150 mEq diluted in 1000 ml of dextrose saline 5% infusing, intravenously, initially 3.5 ml/kg per hour for the first hour before injecting the contrast and following with 1.18 ml/kg per hour during the procedure and for 6 hours after the exam.

Saline solution (0.9%): 1 ml/kg/h intravenously one hour before, during the procedure and extending to 6 hours after contrast administration. All patients will receive this infusion. The patients will be instructed to drink fluids whenever they are thirsty and should not use dopamine, theophylline, furosemide or mannitol two days before nor throughout all the study period.

The medications will be stopped if patients present adverse effects such as severe abdominal pain, bronchospasm, fluid overload determining pulmonary congestion, important basic acid disorder clinically manifested by irritability, neuromuscular excitability and tetany, and cardiac arrhythmia.

Serum creatinine will be performed trough the Jaffe method and cystatin C will be performed trough immunonephelometry using Kits supplied by Dade-Behring, Brazil. The measurements will be taken prior to the examination, 24, 48 and 72 hours after contrast administration. Laboratory tests will be made in a single laboratory.

EACH PATIENT WILL CONTINUE BEING MONITORED BY HIS DOCTOR DURING THE STUDY PERIOD BUT THE RESEARCHER WILL FOLLOW HIM UP WITHIN THE FIRST 72 HOURS ACCORDING TO THE PROTOCOL FOR BLOOD COLLECTION FOR ANALYSIS. THE PATIENTS MAY KEEP CONTACT WITH THE RESEARCHER BY THE PHONES PROVIDED AND BE SEEN IN THE OUTPATIENT NEPHROLOGY SERVICE OF HFA.

THE CRITERIA TO SUSPEND OR TERMINATE THE RESEARCH DURING THE STUDY ARE THE FOLLOWING FACTS: INTERRUPTION IN THE SUPPLY OF THE DRUGS BY THE MANUFACTURER EITHER FOR LACK OF RAW MATERIALS OR FOR THE WITHDRAWAL OF THE DRUG FROM THE MARKET; RISKS OR IMMINENT HARM TO THE PATIENT; OBSERVATION OF THE EXCELLENCE OF ONE DRUG OVER ANOTHER IF TAKEN INTO CONSIDERATION A SIGNIFICANT CASUISTRY.

THE CRITERION FOR THE DEFINITION OF REDUCTION IN RENAL FUNCTION INDUCED BY CONTRAST WILL BE THE INCREASE IN SERUM CREATININE CONCENTRATION OF AT LEAST 0.5 MG PER DECILITER OR CYSTATIN C ABOVE THE REFERENCE RANGE (0.53 TO 0.95MG/L) 48 TO 72 HOURS AFTER CONTRAST ADMINISTRATION.

OTHER RELEVANT ANALYSIS ON RISK FACTORS OF CONTRAST-INDUCED NEPHROPATHY PREDICTORS CAN BE MADE – AGE, VOLUME OF CONTRAST, THE PRESENCE OF DIABETES MELLITUS, AMONG OTHERS.

THE RESEARCHER WILL COORDINATE ALL PATIENT DATA OBTAINED DURING THE STUDY. ANALYZE THEM, CONCLUDE THEM AND MAKE THEM PUBLIC, WHETHER FAVORABLE OR NOT, WITH THE INTENT TO PRESENT THEM TO INTERNATIONAL PUBLICATION.

STATISTICAL ANALYSIS:

TO ESTIMATE THE SIZE OF THE SAMPLE CALCULATION WE USED THE FORMULA NO = 1 / (EO) 2 WITH AN ERROR OF 5%.

THE COLLECTED DATA WILL BE ANALYZED THROUGH THE ANALYSIS OF VARIANCE (ANOVA) OF THE SPLIT-PLOT TYPE (SPANOVA).

A PRELIMINARY ANALYSIS WILL BE PERFORMED WHEN IT IS ACHIEVED THE RESULT OF THE FIRST 100 PATIENTS SINCE THIS SAMPLE ENSURES THE NORMALCY OF EACH GROUP FOLLOWING THE ASSUMPTION OF THE ANALYSIS.

ETHICAL ASPECTS:

THE RESEARCH WILL FOLLOW THE ETHICAL PRINCIPLES ESTABLISHED BY THE 196/96 - RESOLUTION OF THE NATIONAL COUNCIL OF HEALTH. A TERM OF INFORMED CONSENT WILL BE REQUESTED FOR EACH INDIVIDUAL PATIENT BEFORE THEIR INCLUSION IN THE PROTOCOL.

INSTITUTO DO CORÇÃO DO DISTRITO FEDERAL

Proposed appropriation of funds for medical research and/or experimental

Process InCor-DF n.________________

Name of the Principal Investigator: Antonio Jose de Almeida Inda Filho

BUDGET ***FORM 3*** **PAGE N. /**

MATERIAL OF CONSUMPTION TO BE PURCHASED IN BRAZIL (MCB)

ITEM	QUANTITY	DESCRIPTION	UNIT PRICE R$	COST OF THE ITEM R$
01	800	SALINE SOLUTION 0.9%	1,76	1408,00
02	200	DEXTROSE SALINE 5%	2,04	408,00
03	11000	FLUIMUCIL AMPOULES	1,18	12900,00
04	3000	SODIUM BICARBONATE AMPOULES 8.4%	0,47	1410,00
05	1300	EQUIPO FOR INFUSION PUMP	19,00	24700,00
06	1600	TUBE FOR COLLECTING EXAM	0,77	1232,00
07	1200	SYRINGE 10 ML	0,35	420,00
08	400	TELEBRIX – VIALS WITH 100 ML	35,00	14000,00
TOTAL (MCB)				56478,00

FILL IN AS MANY PAGE FORMS AS NECESSARY

INSTITUTO DO CORÇÃO DO DISTRITO FEDERAL

Proposed appropriation of funds for medical research and/or experimental

Process InCor-DF n.________________

Name of the Principal Investigator: Antonio Jose de Almeida Inda Filho

BUDGET ***FORM 7*** **PAGE N. /**

TRANSPORT COSTS (DET)

ITEM	QUANTITY	DESCRIPTION	UNIT PRICE R$	COST OF THE ITEM R$
01	2400	TRANSPORT OF MATERIAL (BLOOD) FOR LABORATORY TESTS	4,58	10992,00
TOTAL (DET)				10992,00

INSTITUTO DO CORÇÃO DO DISTRITO FEDERAL

Proposed appropriation of funds for medical research and/or experimental

Process InCor-DF n.________________

Name of the Principal Investigator: Antonio Jose de Almeida Inda Filho

BUDGET ***FORM 8*** **PAGE N. /**

LIST OF LABORATORY TESTS TO BE PERFORMED (ELA)

ITEMS	DESCRIPTION OF THE TEST	NUMBER PER PATIENT	COST PER PATIENT (R$)	TOTAL NUMBER	TOTAL COST (R$)
01	CREATININE	04	20,00	400	8000,00
02	CYSTATINE C	04	128,00	400	51200,00
03	SODIUM	01	3,50	400	1400,00
04	POTASSIUM	01	3,50	400	1400,00
05	CHLORINE	01	3,50	400	1400,00
TOTAL (ELA)					63400,00

FILL IN AS MANY PAGE FORMS AS NECESSARY
